# Epilepsy and Molecular Phenotype Affect the Neurodevelopment of Pediatric Angelman Syndrome Patients in China

**DOI:** 10.3389/fpsyt.2022.886028

**Published:** 2022-04-28

**Authors:** Shuang Li, Yu Ma, Tianqi Wang, Huimin Jin, Xiaonan Du, Yi Wang

**Affiliations:** ^1^Department of Neurology, Children's Hospital of Fudan University, Shanghai, China; ^2^Shanghai YangZhi Rehabilitation Hospital, School of Medicine, Tongji University, Shanghia, China

**Keywords:** Angelman syndrome, neurodevelopment, GMDS-C, children, epilepsy

## Abstract

**Objective:**

This study investigated the mental development of children with Angelman syndrome (AS) in China and evaluated the relationship between neurodevelopment and molecular subtype, age, epilepsy, and sex using the Chinese version of the Griffith Mental Development Scale (GMDS-C) to provide detailed baseline data regarding neurodevelopment with AS in China.

**Methods:**

Participants were recruited from the AS Natural History Study. The GMDS-C was used to evaluate all participants' mental age and developmental quotients. The general quotient (GQ) and quotients of five subscales (sports, personal-social, auditory language, eye-hand coordination, and comprehensive performance) were calculated.

**Results:**

A total of 119 children (average age: 42.12 months; range, 7.5–95.5 months) with a genetic diagnosis of AS were enrolled. The median GQ score of the GMDS was 29.6 points (95% confidence interval, 28.6–33.25). The children had relatively good locomotor and personal-social skills but poor language skills. Overall, 89% (106/119) had mental ages younger than 24 months for all five subscales. The non-deletion group (i.e., without deletion in chromosome 15q11–13) had higher GQs and locomotor, personal-social, and performance subscale quotients. The GQ was significantly different among the three age subgroups and significantly correlated with age. Compared with the non-epilepsy group, the epilepsy group had lower GQs and lower quotients for the locomotor, personal-social, speech, language, and eye-hand coordination subscales.

**Conclusion:**

Children with AS in China experience severe neurodevelopmental deterioration. In addition to age, molecular subtypes and the onset of seizures may also correlate with these patients' intellectual development. The GMDS-C is an accurate tool that can assess the clinical characteristics of AS. The data of this study can be used as baseline data for clinical trials performed to evaluate drug development or other AS treatment development.

## Introduction

Angelman syndrome (AS; OMIM: 105830) is a rare neurogenetic disorder prevalent in approximately 1 in 24,000 to 1 in 52,000 of the population (1, 2). AS is characterized by developmental delay, epilepsy, loss of language, and the tendency to laugh (3). Although the clinical features and neurodevelopment vary, all patients require lifelong care, thereby imposing a huge burden on the family and society (4, 5). Etiologically, AS involves decreased expression of maternal ubiquitin ligase protein 3a (UBE3A), which has maternal-specific expression in neurons by genomic imprinting (6, 7). The four molecular subtypes of AS have different mechanisms: maternal deletion in 15q11–q13, which involves the critical region (68–75%); mutation in *UBE3A* (Mut) (8–11%); paternal uniparental disomy (UPD) (2–7%); and imprinted gene defect (ID) (2–5%) (8, 9).

Children with AS usually have a considerable intellectual disability, especially language delay, and the few patients who can express themselves have limited vocabulary (10). Additionally, patients with AS have movement incongruity, dystonia, and other conditions that limit motor development (11). These characteristic developmental delays and imbalances can be used to diagnose AS.

The molecular subtype affects the phenotype, and individuals with deletions in 15q11–13 often have a more severe presentation than those without deletions (12, 13). A study conducted in 2020 found that compared with the non-deletion type, children with AS of deletion type had lower scores at baseline and lower acquisition of skills using the Bayley-III. This study also simultaneously describes the developmental trajectories of individuals of all molecular subtypes (14). Epilepsy may also affect the development of AS. Epilepsy occurs in approximately 80% of children with AS, and the first epileptic attack usually occurs early, before age 3 years (15). However, although there have been studies on the mechanism of and treatment for epilepsy in children with AS (6, 16, 17), the relationship between epilepsy and intellectual development has not been clarified. One small study found that epileptic activity is not associated with developmental milestones in patients with AS (18), while another study suggested that early-onset epilepsy (<2 years of age) may cause severe development delay (19). Given that the current conclusions are still controversial, we also took this factor into account in this study. Otherwise, the effect of sex on neurodevelopment has not been studied.

To the best of our knowledge, despite the increasing number of AS cases in China (20, 21), no studies have examined their neurodevelopment objectively. Additionally, although natural history research has become increasingly important to targeted therapy development, the neurodevelopment of patients with AS is often difficult to evaluate and compare because of the severe intellectual disability and dispersion (22). Furthermore, current intelligence screening methods have many limitations. The Wechsler Intelligence Scale (23) could accurately identify children with intellectual development disorder, but it lacks discrimination for diseases involving severe intellectual disability. Bayley-III, which is a scale designed for children younger than 42 months (24, 25), has limited potential for evaluating developmental skills; therefore, it may fail to appreciate the skills of children with advanced development. Although Bayley-III has been used to assess the development of patients with AS, there is still no consensus regarding the appropriate threshold for neurodevelopmental delay (26, 27). Because of the lack of standardized guidelines for the use of Bayley-III in China, the long evaluation time, and the complicated evaluation and analysis, it is not considered well-suited for assessing children with AS. The Gesell Developmental Scale, which is suitable for children younger than 6 years, has similar limitations and has not been revised in the Chinese context for more than 20 years (28).

The Griffiths Mental Development Scale Chinese version (GMDS-C) is an accurate and objective tool used to assess the mental development of children from birth to 8 years, especially those with developmental disorders (29). It is widely used in many countries and has excellent psychometric performance (30, 31). The GMDS-C was improved and updated in the Chinese context in 2016. An across-context analysis also confirmed that the GMDS is applicable for the assessment of children from birth to age 8 years in China (32). Furthermore, language skills do not have much influence on the evaluation of other scales, thereby allowing children with AS to perform more tasks despite the lack of language.

This study aimed to investigate the mental development of children with AS in China and evaluate the correlation between neurodevelopment and the molecular subtype, age, epilepsy, and sex using the GMDS-C. We collected the clinical and genetic characteristics of 119 children with genetically confirmed AS and evaluated their locomotor, personal-social, speech, language, and eye-hand coordination abilities using the GMDS-C. Furthermore, the age at the time of diagnosis of AS was collected, and the mental age, subscale quotient, and general quotient (GQ) were calculated as statistical indicators for analysis (33, 34) to provide detailed neurodevelopmental baseline data for AS natural history research in China and provide evidence for follow-up intervention research.

## Methods

### Study Design and Participants

This cross-sectional study was approved by the Ethical Institutional Review Committee of Children's Hospital of Fudan University and was conducted according to the tenets of the Declaration of Helsinki. Written informed consent was obtained from the legal guardian of each participant.

Participants in the AS Natural History study (ClinicalTrial.gov identifier: NCT03358823) were studied from January 2015 to December 2020. At least two pediatric neurologists at the Children's Hospital of Fudan University in China that were familiar with AS, conducted interviews and physical examinations of all patients. The inclusion criteria were as follows: age 6 months to 8 years; molecularly confirmed diagnosis of AS that can be classified as one of the four molecular subtypes (maternal deletion in the key region of 15q11.2–q13; paternal UPD; imprint center defect [ID]; and *UBE3A* mutation); clinical characteristics corresponding to the updated consensus for the diagnostic criteria of AS in 2005 (3); and no other comorbidities that might obscure the AS phenotype. Participants were excluded if they had the mosaic genotype or frequent epileptic attacks that may affect the evaluation process.

Participants with a deletion in chromosome 15q11–13 were classified as the deletion group, whereas participants with the other three molecular subtypes were classified as the non-deletion group. The deletion group was further divided into three subgroups by age as follows: group 1, age 6–36 months; group 2, age 36–60 months; and group 3, age 60–96 months. To analyze the correlation between neurodevelopment and epilepsy, age-matched patients with and without epilepsy were categorized as the epilepsy group and non-epilepsy group; all of those patients with and without epilepsy were in the deletion group.

### Neurodevelopmental Assessment

General characteristics, including age, sex, mutation type, age at the time of the first evaluation, age at the time of diagnosis, and seizure status (according to the International League Against Epilepsy Classification of the Epilepsies in 2017) (30) were collected using a self-designed general survey form. Neurodevelopment was assessed using the GMDS-C. Briefly, the GMDS-C includes six subscales (locomotor, personal-social interaction, hearing and language, eye-hand coordination, performance, and practical reasoning); only five of those subscales (locomotor, personal-social interaction, hearing and language, eye-hand coordination, performance) are needed to evaluate children younger than 2 years. The development quotient (DQ) was expressed as the ratio of the DQ as follows: DQ = (mental age/chronological age) × 100. We calculated subscale quotients for each subscale and one GQ for general. The mean GQ was 100 in normal children (standard deviation [SD] = 15), and a quotient <70 for each subscale indicated severe neurodevelopmental delay (23, 24).

### Statistical Analysis

The results were analyzed according to different molecular subtypes, presence of epilepsy, and age at the time of assessment. The Kolmogorov-Smirnov test was used to analyze data normality. Continuous variables are described as mean ± SD or the 50th percentile (median) with the 25th percentile and 75th percentile; categorical variables are presented as the frequency and percentage. A *t*-test or non-parametric test was used to compare differences between two groups; a one-way analysis of variance was applied to compare more groups. The association of age with the subscale quotients and GQs was investigated using a linear analysis. All statistical analyses were performed using SPSS Statistics (version 20.0; IBM Corp., Armonk, NY, USA). The significance threshold was set at *P* < 0.05.

## Results

### Neurodevelopment

A total of 119 children (75 boys and 44 girls) with a mean age of 42.12 months (SD = 21.0; range, 7.5–95.5 months) were enrolled. The median GQ was 29.6 points (95% confidence interval = 28.6–33.25 points). None of the participants had measurable scores for practical reasoning, poor language skills, and limited developmental levels. In addition, there was a significant difference in the five subscale quotients (*P* < 0.0001). Table 1 shows the subscale quotients and GQs of the participants. A comparison of the subscale quotients with GQs showed that the quotient of the locomotor subscale was significantly higher than the GQ (*P* = 0.0061), whereas the quotient of the language subscale was significantly lower than the GQ (*P* < 0.0001).

**Table 1:** Results of GMDS-C assessment^a^.

	**Quotient^b^**	**Severe delay *n* (%)^b^**	***P-*value^c^**
Locomotor subscale	33.8 [27.06, 45.45]	113 (95.0%)	0.0061^**^
Personal-social subscale	32.35 [22.96, 42.86]	115 (96.6%)	0.6878
Language subscale	22.06 [14.84, 29.33]	117 (98.3%)	<0.0001^**^
Eye-hand coordination subscale	29.41 [20.64,37.51]	117 (98.3%)	>0.9999
Performance subscale	28.04 ± 11.9	118 (99.2%)	0.7252
GQ	29.6 [21.54, 37.69]	118 (99.2%)	1

a *n = 119*.

b *Non-normally distributed data are described as the median [25th percentile, 75th percentile], and normally distributed data are described as the mean ± standard deviation (SD)*.

b *Severe delay is defined as a general quotient (GQ) or subscale quotient < 70 (-2 SD)*.

c *Comparison between subscale quotients and GQs; values in bold are statistically significant (*

** *P < 0.01)*.

**Figure 1:**
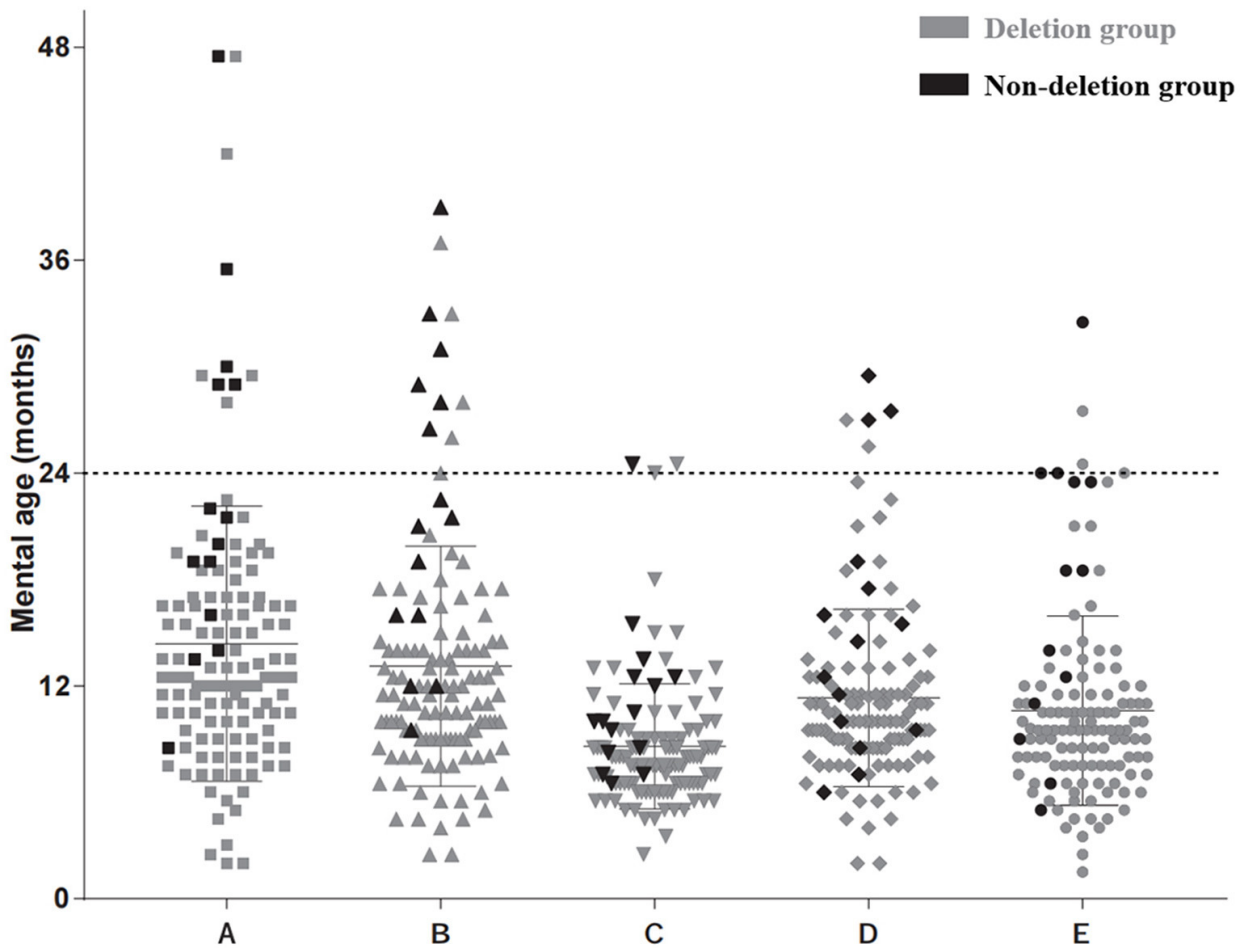
Mental age distribution according to five subscales. **(A)** Locomotor subscale. **(B)** Personal-social subscale. **(C)** Speech and language subscale. **(D)** Eye-hand coordination subscale. **(E)** Performance subscale. Each black dot represents one result of the deletion type in this area. Each gray dot represents one result of the non-deletion type.

Regarding mental age, 89.1% (106/119) of the participants had mental ages younger than 24 months for all five subscales, and the other 11.9% (13/119) had mental ages older than 24 months for at least one subscale (seven patients were in the deletion group and six patients were in the non-deletion group). The distribution of mental ages for the five subscales differed and ranged from 2 months to 47.5 months for the locomotor subscale; from 5 months to 39 months for the personal-social subscale; from 2.5 months to 24.5 months for the speech and language subscale; from 2 months to 36 months for the eye-hand coordination subscale; and from 1.5 months to 40 months for the performance subscale. The specific distributions are shown in Figure 1.

### Distribution of the Subscale Quotients by Age

**Figure 2:**
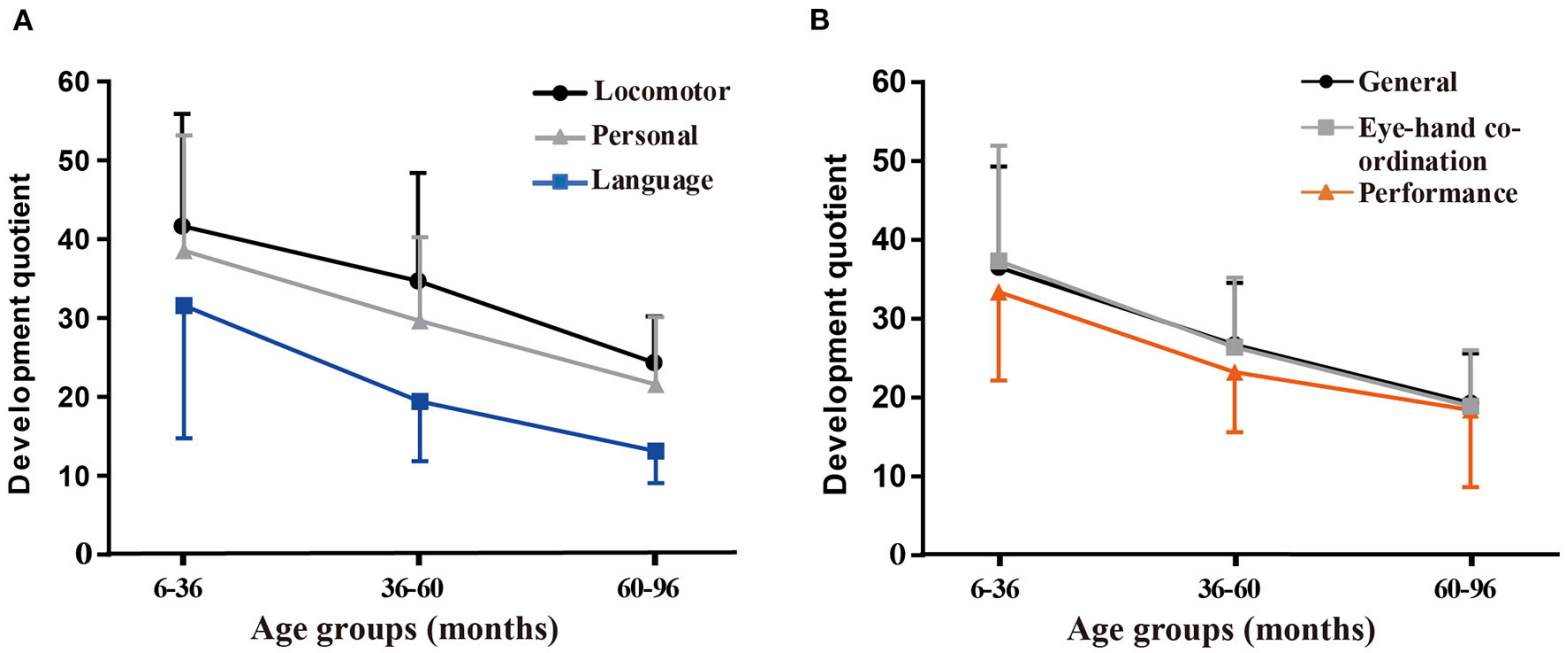
Variations in the mean general quotients (GQs) and quotients for the five subscales according to the age group. **(A)** Variations in the mean quotients for the locomotor, personal-social, and language subscales. **(B)** Variations in the mean GQs and quotients for hand-eye coordination and performance subscales.

Table 2 shows the distribution of GQs and subscale quotients of the participants in the following age groups: 6–36 months (48 observations); 36–60 months (34 observations); and 60–96 months (21 observations). There were significant differences in GQs and all subscale quotients among the three age subgroups (*P* < 0.001). A correlation analysis of different age subgroups found significant negative correlations between the chronological age during assessment and the development quotients for the five subscales and GQs (*r* < 0; *P* < 0.001). The SDs of the mean are shown by the error bars in Figure 2.

**Table 2:** Distribution of the subscale quotients for the GMDS-C by age group^a^.

	**Age 6–36 months** **(*n* = 48)**	**Age 36–60 months** **(*n* = 34)**	**Age 60–96 months** **(*n* = 21)**	***r***	***P-*value^b^**
Locomotor subscale	38.12 [33.3, 49.52]	30.59 [27.11, 38.68]	24.26 ± 5.84	−0.441	<0.001^**^
Personal-social subscale	35.53 [29.72, 44.29]	26.88 [22.48, 35.93]	19.1 [15.87, 26.17]	−0.510	<0.001^**^
Language subscale	26.52 [22.92, 32.47]	16.94 [14.24, 22.77]	13.1 ± 4.06	−0.623	<0.001^**^
Hand-eye coordination subscale	34.87 [27.07, 44.23]	25.02 [20.47, 29.55]	17.83 [13.02, 22.63]	−0.629	<0.001^**^
Performance subscale	33.28 ± 11.21	23.09 ± 7.59	16.94 [12.56, 20.84]	−0.587	<0.001^**^
GQ	32.97 [28.94, 40.74]	24.18 [21.07, 30.33]	19.17 ± 6.30	−0.617	<0.001^**^

a *Non-normally distributed data are described as the median [25th percentile, 75th percentile], and normally distributed data are described as the mean ± standard deviation*.

b *Values in bold are statistically significant (*

** *P < 0.01)*.

*GQ, general quotient*.

### Correlation Between Neurodevelopment and Molecular Subtypes

Among all the participants, 88.2% (105/119) had a chromosome 15q11–q13 deletion, 5.04% (6/119) had UPD, 7.56% (9/119) had a *UBE3A* variant, and 0.8% (1/119) had ID. Table 3 shows the distribution of GQs and the subscale quotients by molecular subgroup. The non-deletion group had higher GQs and quotients for the locomotor, personal-social, and performance subscales (all *P* < 0.05); however, there was no significant difference in the quotients for the language and eye-hand coordination subscales (all *P* > 0.05).

**Table 3:** Correlation between neurodevelopment and molecular subtypes^a^.

**Variable**	**Deletion^b^** **(*n* = 103)**	**Non-deletion^c^** **(*n* = 16)**	**Z/t score**	***P-*value^d^**
**Age (months)**	37.91 [26.00, 56.61]	37.75 [24.89, 67.06]	0.306	0.760
Age at first visit	10.73 ± 5.13	13.27 ± 7.66	9.391	<0.001
Age at diagnosis	23.72 ± 12.99	25.27 ± 12.23	2.604	0.026
**Subscales:**				
Locomotor	32.62 [26.3, 41.2]	45.09 [36.48, 62.97]	1.707	0.006^**^
Personal-social	30.27 [21.65, 40.45]	49.11 [33.41, 65.82]	3.318	0.001^**^
Language	21.84 [14.61, 26.67]	24.93 [18.51, 36.87]	1.831	0.067
Eye-hand coordination	29.82 [21.62, 35.93]	36.15 [25.33, 42.12]	1.788	0.074
Performance	24.93 [18.83, 34.15]	37.84 [29.58, 43.46]	3.030	0.002^**^
GQ	28.92 [21.2, 34.44]	38.29 [30.62, 50.5]	1.852	0.002^**^

a *Non-normally distributed data are described as the median [25th percentile, 75th percentile], and normally distributed data are described as the mean ± standard deviation*.

b *Deletion group: participants with a deletion on chromosome 15q11-13*.

c *Non-deletion: participants with uniparental disomy, imprinted gene defect, or UBE3A mutation*.

d *Values in bold are statistically significant (*

** *P < 0.01)*.

*GQ, general quotient*.

### Correlation Between Neurodevelopment and Epilepsy

During this study, 79.8% (95/119) of patients reported clinical seizures. We selected 30 age-matched patients without epilepsy. As shown in Table 4, there were differences in DQs among four subscales (i.e., locomotor scale, personal-social scale, speech and language, and eye-hand coordination) and GQs (all *P* < 0.05). There was no significant difference in the quotients for the performance subscale. The parents of six children reported developmental regression after the seizure; this regression was reflected in the movement of five children.

**Table 4:** Correlation between neurodevelopment and epilepsy^a^.

**Variable**	**Epilepsy^b^** **(*n* = 30)**	**Non-epilepsy^c^** **(*n* = 24)**	***T* score**	***P-*value^d^**
**Chronological age (months)**	27.39 ± 5.12	27.26 ± 17.31	0.033	0.974
**Subscales:**				
Locomotor	38.04 ± 9.75	43.92 ± 18.51	1.257	0.219
Personal-social	33.01 ± 8.21	43.16 ± 19.16	2.178	0.039*
Language	24.85 ± 5.18	38.43 ± 24.19	2.452	0.023*
Eye-hand coordination	33.70 ± 6.63	44.40 ± 19.63	2.311	0.030*
Performance	30.51 ± 6.44	36.85 ± 15.21	1.717	0.098
GQ	32.02 ± 5.54	41.35 ± 17.28	2.300	0.031*

a *Non-normally distributed data are described as the median [25th percentile, 75th percentile], and normally distributed data are described as the mean ± standard deviation*.

b *Epilepsy group: participants with epileptic seizures at least once before assessment*.

c *Non-epilepsy group: participants without any epileptic seizure before assessment*.

d Values in bold are statistically significant (*P < 0.05).

*GQ, general quotient*.

### Correlation Between Neurodevelopment and Sex

The correlation between neurodevelopment and sex for participants in the deletion group was analyzed to rule out the influence of the molecular type. As shown in Table 5, there was no significant difference in neurodevelopment between the two sex groups.

**Table 5:** Sex differences in developmental levels^a^.

	**Boys** **(*n* = 66)**	**Girls** **(*n* = 37)**	***T* score**	***P-*value**
Age (months)	43.39 ± 21.47	40.26 ± 20.53	0.805	0.422
**Subscales:**				
Locomotor	35.49 ± 13.66	41.08 ± 19.31	0.068	0.068
Personal-social	33.02 ± 14.22	36.8 ± 18.78	0.217	0.217
Language	22.81 ± 10.94	22.62 ± 18.08	0.155	0.155
Eye-hand coordination	29.89 ± 12.38	31.27 ± 15.5	0.593	0.593
Performance	28.01 ± 11.5	28.09 ± 12.69	0.971	0.971
GQ	29.84 ± 11.15	32.77 ± 15.14	0.22	0.229

*^a^Non-normally distributed data are described as the median [25th percentile, 75th percentile], and normally distributed data are described as the mean ± standard deviation*.

*GQ, general quotient*.

## Discussion

The neurodevelopment of patients with AS is often difficult to evaluate; therefore, its characteristics and influencing factors have not been completely elucidated. The results of this study indicated that children with AS in China experience severe neurodevelopmental deterioration. In addition to age, molecular subtypes and the onset of seizures also correlate with the intellectual development of these patients. To the best of our knowledge, this is the largest cross-sectional study to use the GMDS-C to assess the development of children with AS; furthermore, it is also the first to collect objective developmental data of AS patients in China. Our data showed that 99.2% of the participants had GMDS-C GQs <70, thus indicating severe neurodevelopmental delay. Furthermore, the 95% confidence interval of the GMDS-C GQ ranged from 28.6 to 33.25 points. Such low values generally do not appear with developmental delays caused by exogenous factors, such as premature birth, infection, trauma, and extreme malnutrition (35, 36). Additionally, similar to the findings of other studies (5, 37), there was an imbalance in cognitive development; the participants had relatively strong locomotor and personal-social skills but weak language skills. These findings indicate that clinicians should be alerted to consider a diagnosis of AS if the patient has a declining GQ of ~29.6 points according to the GMDS-C, has good locomotor and personal-social skills, and has poor language skills.

Regarding the developmental age, 88.4% of the participants had a developmental age within 24 months, whereas the other 11.6% had a developmental age older than 24 months for at least one subscale; the highest developmental age was 47.5 months (observed using the locomotor scale). This age is older than that observed during a 2001 study that also used the GMDS to assess 20 children with AS (age 2–14 years) (38) and reported that all the participants had developmental ages younger than 24 months. This difference may reflect an improvement in the understanding of AS; previous diagnoses and rehabilitation may increase the upper limit of the developmental level, which needs to be confirmed by further research. Moreover, the results may vary by the tool used for the evaluation, the population, and the culture (31). A 2021 study that used Bayley-III showed that children with AS demonstrated skills when their developmental age was approximately 14 to 27 months, but their actual age was 6 years (14). Language skills are not a major factor in the GMDS-C assessment, thus allowing children with AS to perform more tasks despite their lack of language skills. The clinical symptoms of children with AS are mainly caused by the *UBE3A* gene loss of function, which has been well-confirmed in mouse models (39). The pathogenesis of AS is unclear, while diffusion tensor imaging of the brain has indicated defects in language pathways (40, 41), and motor impairments may be associated with cerebellar dysfunction and defects in motor cortex and nigrostriatal pathways (42, 43).

A linear analysis indicated an overall reduction in the GQ and quotient for all subscales with increasing age, indicating the progressive neurodevelopmental deterioration of children with AS, consistent with clinical observations (43, 44). The reduction was more obvious in the younger group and was decreased in the older group. Although we did not include patients older than 8 years, the downtrend also indicates changing trends, thus helping to predict the subsequent development of AS. It seemed that children with AS continued to develop higher skill levels over time, but at a slower rate. A 2021 study showed that the developmental skills of children with AS continued to improve until at least 12 years of age (14).

We verified that individuals with deletions in the 15q11–13 chromosome experience worse development, consistent with the results of prior studies (45). A non-parametric test showed lower GQs and quotients for sports, personal-social, and performance subscales in the deletion group than in the non-deletion group. This difference may be attributable to the deficiency of GABA_A_ receptor subunit genes in the 15q11–13 region other than *UBE3A*, which has been associated with epilepsy and developmental delay (46). Interestingly, the two molecular subgroups did not show significant differences in language and eye-hand coordination. However, we believe that if the sample size had been sufficiently large, then the scores for all subscales would have been significantly different between the two groups. Nevertheless, it still suggested that the scores of these two subscales should not be used as criteria to distinguish between the two molecular subgroups. A study of 22 individuals with mosaic AS showed that the most prominent characteristic is the preservation of the ability to express language (47), indicating that UBE3A may be crucial to the development of brain regions that control this ability.

In addition to the molecular subtype, epilepsy, which is a common symptom of AS, also influences the cognition of patients with AS. The *t*-test results showed that the epilepsy group had worse GQ and quotients for the locomotor, personal-society, eye-hand coordination, and performance subscales. Additionally, five of our participants developed significant regression of development milestones after epileptic seizures; however, their abilities gradually improved after the seizures were controlled. This is intuitive evidence that epilepsy may affect the development of children with AS. Although there is currently no radical treatment for AS, therapies focused on reducing the severity of seizures and minimizing their frequency could be beneficial for the neurodevelopment of children with AS (16, 48). A 2008 study showed that one-fourth of patients with epilepsy have ID, and that one-fifth of patients with ID have epilepsy (49). Therefore, further research is needed to clarify whether worse development itself is a high-risk factor for seizures occurring in patients with AS.

Although AS was first described in 1965, the few descriptive studies performed in China include small samples (20, 50). The interval between diagnosis and the first evaluation was almost 18 months during this study, which is unfavorable for early rehabilitation and family genetic counseling. Many studies have explored different methods of gene therapy for curing AS (51, 52). Regarding assessments, the GMDS-C is more suitable because it can be applied to a broader age range, provides a detailed assessment of different aspects of development, has low reliance on language, and can be adapted to the Chinese culture. Therefore, the findings of this study provide basic evidence for the use of the GMDS-C to establish the baseline natural history of AS.

This study had some limitations. The use of a cross-sectional design made it difficult to represent the development trends of the same groups according to age. Additionally, controlled trials and a longitudinal cohort study are needed to provide clinical guidance. Furthermore, the number of participants in the non-deletion group was limited, thus making it difficult to compare different subtypes (*UBE3A* mutation, UPD, and ID) in this group; however, this will be an important focus of our research in the future.

In conclusion, our study uses the GMD-S scale, which is well-established in China, to fill the gap in the assessment of the baseline level of the natural developmental history of AS. It was confirmed that molecular subtypes and age influence the intellectual development of children with AS, and our results further indicated that epileptic seizures cause secondary developmental regression; therefore, epilepsy control is important for development. These results may provide useful endpoints for clinical trials that can be performed to evaluate the development of drugs and other treatments for AS.

## Data Availability Statement

The original contributions presented in the study are included in the article/supplementary material, further inquiries can be directed to the corresponding author/s.

## Ethics Statement

The studies involving human participants were reviewed and approved by Ethical Institutional Review Committee of Children's Hospital of Fudan University. Written informed consent to participate in this study was provided by the participants' legal guardian/next of kin.

## Author Contributions

SL, XD, and YW contributed to developing the concept of the manuscript. SL wrote the first draft of the manuscript. SL, XD, and HJ participated in the Griffith assessments. YM and TW participated in the collection and analysis of data. All authors contributed to manuscript revision, read, and approved the submitted version.

## Funding

This work was supported by the Shanghai Municipal Science and Technology Major Project (Grant No. 2017SHZDZX01), Shanghai Key Laboratory of Birth Defects, Shanghai, 201102, China (Grant No. 13DZ2260600), Shanghai Municipal Science and Technology Major Project (Grant No. 2018SHZDZX03), and Omics-based precision medicine of epilepsy being entrusted by Key Research Project of the Ministry of Science and Technology of China (Grant No. 2016YFC0904400).

## Conflict of Interest

The authors declare that the research was conducted in the absence of any commercial or financial relationships that could be construed as a potential conflict of interest.

## Publisher's Note

All claims expressed in this article are solely those of the authors and do not necessarily represent those of their affiliated organizations, or those of the publisher, the editors and the reviewers. Any product that may be evaluated in this article, or claim that may be made by its manufacturer, is not guaranteed or endorsed by the publisher.
